# Epigenetic Signatures of Centrosomes Are Novel Targets in Cancer Diagnosis: Insights from an Analysis of the Cancer Genome Atlas

**DOI:** 10.3390/epigenomes6020014

**Published:** 2022-06-02

**Authors:** Zhou Zhang, Wei Zhang

**Affiliations:** 1 Department of Preventive Medicine, Northwestern University Feinberg School of Medicine, Chicago, IL 60611, USA; zhou.zhang@northwestern.edu; 2 The Robert H. Lurie Comprehensive Cancer Center, Northwestern University Feinberg School of Medicine, Chicago, IL 60611, USA

**Keywords:** centrosome, DNA methylation, TCGA, 450K array, RNA-seq, cancer detection

## Abstract

The centrosome plays a central role for cellular signaling and is critical for several fundamental cellular processes in human cells. Centrosome abnormalities have been linked to multiple solid tumors and hematological malignancies. We sought to explore the potential role of the DNA methylation, a critical epigenetic modification, of centrosome-related genes in different cancers. The 450K array DNA methylation data and RNA-seq data were downloaded for ~4000 tumor samples and ~500 normal controls from The Cancer Genome Atlas (TCGA) project, covering 11 major cancer types. Cancers with more than 30 normal controls were retained for analysis. Differentially modified CpGs of centrosome genes were identified, and cancer-specific epigenetic models were developed using a machine-learning algorithm for each cancer type. The association between the methylation level of differential CpGs and the corresponding gene expression, as well as the co-localization of the differential CpGs and *cis*-regulatory elements were evaluated. In total, 2761 CpGs located on 160 centrosome genes for 6 cancers were included in the analysis. Cancer-specific models demonstrated a high accuracy in terms of the area under the receiver operating characteristic (ROC) curve (AUC > 0.9) in five cancers and showed tissue specificity. This study enhanced our understanding of the epigenetic mechanisms underlying the DNA methylation of centrosome-related genes in cancers, and showed the potential of these epigenetic modifications as novel cancer biomarkers.

## 1. Introduction

The centrosome is a non-membranous organelle comprised of centrioles surrounded by pericentriolar material (PCM) [1,2,3] and over 150 proteins [4,5]. The centrosome functions as the major microtubule organizing center (MTOC) of human cells. The architecture of PCM changes during mitosis with the inner layer expansion and further components addition. Together, their activities result in a mature centrosome with a maximal MTOC activity [6,7]. In human cells, the centrosome consists of a pair of orthogonally positioned centrioles. The mother centriole, the older one of the two centrioles, functions as the fundamental structure that assembles the primary cilium, which plays a central role for cellular signaling [8]. Several fundamental cellular processes, such as polarity and division, are governed by the centrosome [9], indicating its potential role in the pathogenesis of human diseases.

Specifically, centrosome abnormalities have been implicated in solid tumors such as lung, breast, prostate, colon, ovarian and pancreatic cancer [10,11,12,13,14], as well as hematological malignancies such as multiple myeloma, lymphomas, and acute and chronic myeloid leukemia [15,16]. However, how centrosome abnormalities affect tumorigenesis remains largely unknown. Molecularly, centrosome abnormality involves multiple genetic and epigenetic factors. Thus, investigating DNA methylation in centrosomes, where critical epigenetic modifications may contribute to tumorigenesis, would yield novel insights into human cancers [17,18,19,20,21] and provide a new opportunity for cancer diagnosis. Elucidating the association between DNA methylation and centrosome-related genes in cancers, whether direct or indirect, could therefore provide novel insights into the underlying mechanisms of tumorigenesis.

In the current study, we systematically analyzed the DNA methylation data from The Cancer Genome Atlas (TCGA) Project [22], specifically the 450K array data covering 11 major cancer types [23]. A targeted analysis of highly informative CpG sites located in over 150 centrosomal genes [4,5] was conducted. This study helped us understand the epigenetic mechanism underlying the DNA methylation of centrosome-related genes in cancers, and laid the foundation for utilizing these epigenetic modifications as novel cancer biomarkers.

## 2. Results

### 2.1. Identification of Cancer-Specific CpGs

**Figure 1:**
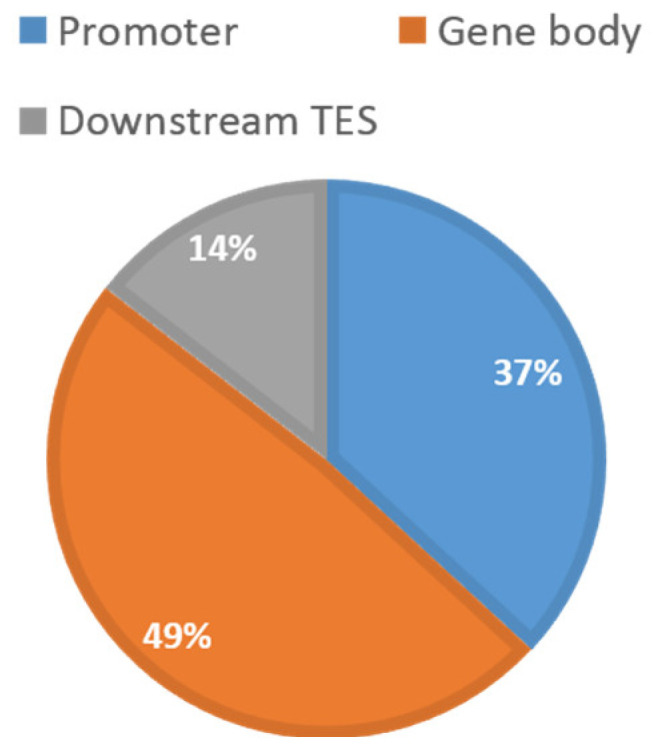
Relative distribution of the 2761 centrosome-related CpGs. All 2761 CpGs located on 160 centrosome genes are grouped by their relative locations to the genic region. Promoter is defined as 10 kb upstream to transcription start site (TSS). Downstream TES was defined as from transcription end site (TES) to 10 kb downstream of the genes. This distribution follows the general distribution of probes from the 450K array.

**Figure 2:**
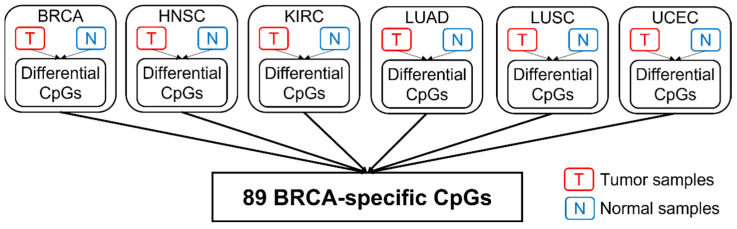
The workflow for identifying cancer-specific CpGs. The detection of BRCA-specific CpGs is shown as an example. First, differential CpGs for each cancer type are identified by comparing tumor samples and normal samples. To obtain cancer-specific CpGs for BRCA, the identified differential CpGs (BRCA) are then mapped to the differential CpGs from other cancer types to identify CpGs unique to BRCA.

Table 1 shows a summary of the available data from TCGA. In total, 2761 CpGs located on 160 centrosome genes were retained for analysis. The relative distribution along the genic region was 37% (promoter), 49% (gene body), and 14% (downstream of TES), respectively (Figure 1). This distribution appeared to follow the general distribution of probes from the 450K array. Table 2 shows the summary of the detected cancer-specific CpGs and genes. In total, 701 CpGs (123 genes) in breast invasive carcinomas (BRCA), 531 CpGs (121 genes) in head and neck squamous cell carcinomas (HNSC), 1283 CpGs (133 genes) in kidney renal papillary cell carcinomas (KIRC), 228 CpGs (87 genes) in lung adenocarcinomas (LUAD), 1366 CpGs (136 genes) in lung squamous cell carcinomas (LUSC), and 489 CpGs (112 genes) in uterine corpus endometrial carcinomas (UCEC) with FDR < 0.05 were identified as being differentially methylated between tumors and corresponding normal controls. Among them, 33 CpGs were shared by all cancer types. After comparing across all cancer types, as described in Figure 2, the number of cancer-specific CpGs was 89 (54 genes) in BRCA, 86 (62 genes) in HNSC, 186 (88 genes) in KIRC, nine (five genes) in LUAD, 320 (110 genes) in LUSC, and 45 (37 genes) in UCEC for each cancer type, respectively.

### 2.2. Cancer-Specific Epigenetic Model

**Figure 3:**
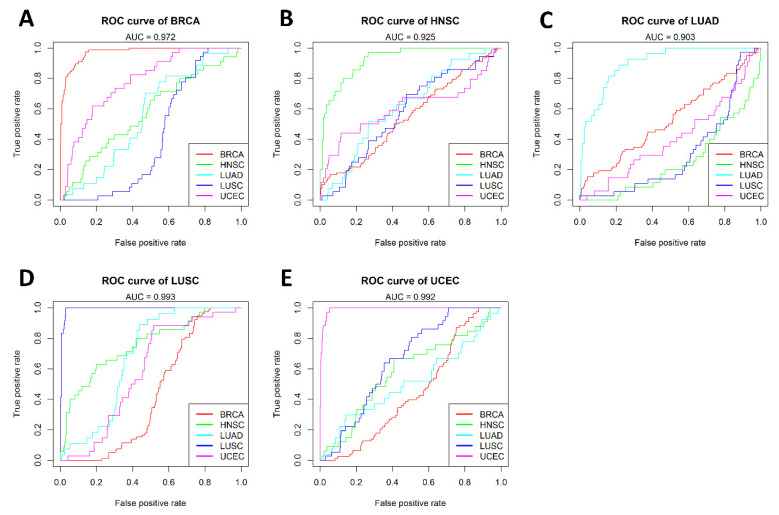
Comparison of model performance across all cancer types. The performance of the five cancer-specific models built using the cancer-specific CpGs for: (**A**) BRCA; (**B**) HNSC; (**C**) LUAD; (**D**) LUSC; and (**E**) UCEC. Each model was also tested for samples from other cancer types, and demonstrated a superior performance for its respective cancer type but a poor performance for other cancer types. Each color line represents a cancer type.

The Support Vector Machine (SVM) [24] was used to build the cancer-specific model for each cancer type. For each model, the number of features (cancer-specific CpGs) was seven (BRCA), five (HNSC), 15 (KIRC), five (LUAD), seven (LUSD), and six (UCEC), respectively. Each cancer-specific model demonstrated a superior performance for its respective cancer type, in contrast to a relatively poor performance for other cancer types. Figure 3 shows the respective AUC for BRCA (0.972; Figure 3A), HNSC (0.925; Figure 3B), LUAD (0.903; Figure 3C), LUSC (0.993; Figure 3D), and UCEC (0.992; Figure 3E) based on the cancer-specific models. While five models achieved an AUC greater than 0.9, the KIRC model only achieved an AUC of 0.523 and was therefore excluded from further analysis.

### 2.3. Tissue Specificity Underlying Cancer Type Specificity

**Figure 4:**
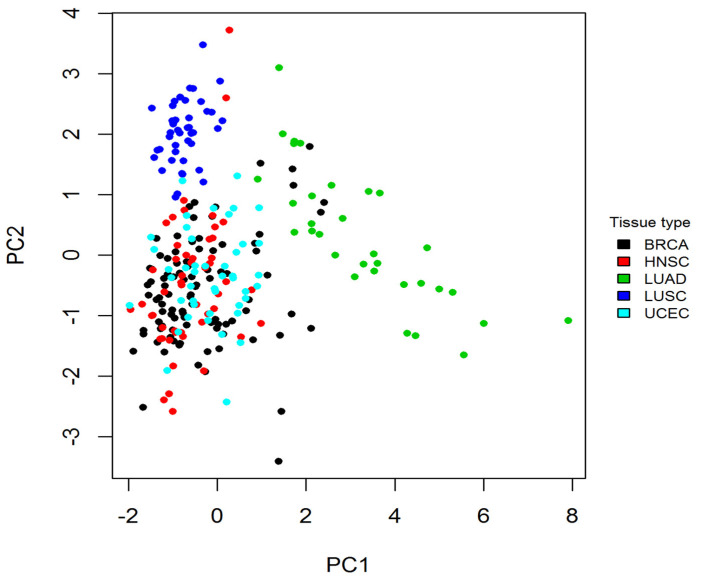
The PCA analysis shows a tissue-specific pattern in normal samples. For normal samples, the 1st and 2nd principal components derived from the methylation levels of cancer-specific CpGs are plotted. Each dot represents a normal sample, and each color represents a tissue type. The plot shows a clear separation between normal samples from LUAD, LUSC, and the combination of BRCA, UCEC, and HNSC. PCA: principal component analysis.

To determine whether tissue specificity caused cancer-specific CpGs, a principal component analysis (PCA) was carried out for the CpGs in normal samples. When plotting the 1st principal component against the 2nd component, a tissue-specific pattern was observed (Figure 4), indicating a clear separation of LUAD, LUSC, and the combined samples of BRCA, UCEC, and HNSC.

### 2.4. Association Analysis between CpG and Gene Expression

The Pearson’s correlation between the methylation level of each CpG site and its local host gene was calculated for each type of cancer in all samples, then separately for tumor and normal samples (Figure S1). In all samples, the mean correlations were −0.02 (BCRA), 0.11 (HNSC), −0.01 (KIRC), −0.06 (LUAD), 0 (LUSC), and −0.04 (UCEC), respectively. In tumor samples, the mean correlations were −0.02 (BCRA), 0.12 (HNSC), −0.01 (KIRC), −0.37 (LUAD), 0 (LUSC), and −0.03 (UCEC), respectively. In contrast, in normal samples, the mean correlations were 0.01 (BCRA), −0.12 (HNSC), −0.05 (KIRC), 0.02 (LUAD), 0.09 (LUSC), and −0.04 (UCEC), respectively. Regardless of the CpG location, such as the promoter and gene body, there were no statistically significant differences between the mean correlations of tumor and normal samples. However, in normal samples, when comparing promoters and gene bodies, the mean correlations of CpGs in promoters were significantly lower than those in the gene bodies of BCRA, HNSC, KIRC, and UCEC (student’s *t*-test, *p*-value < 0.05; Figure S2A). There was no significant difference in tumor samples (Figure S2B).

### 2.5. Co-Localization of Differential CpGs with Cis-Regulatory Elements

**Figure 5:**
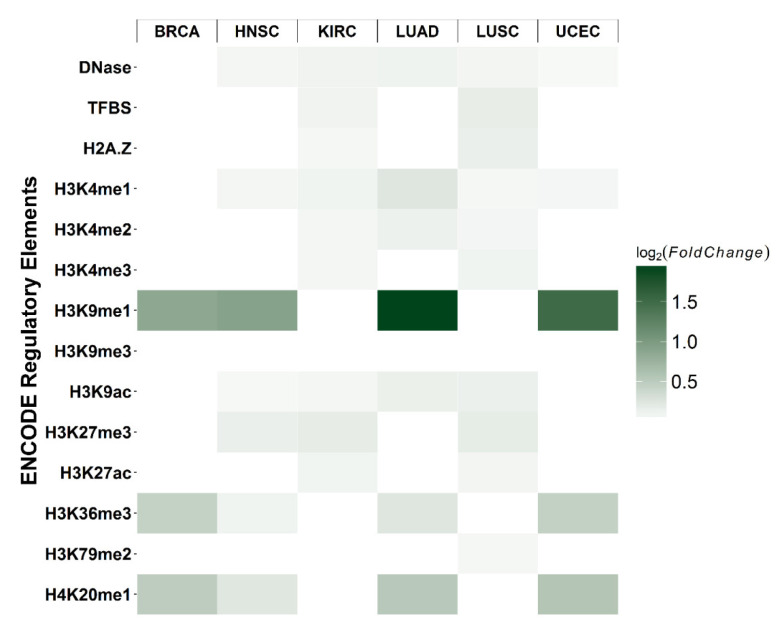
Enrichment of differential CpGs with *cis*-regulatory elements. Each rectangle represents the log_2_ fold-change of observed overlapping between differential CpGs (e.g., BRCA) and a *cis*-regulatory element (e.g., H3K9me3), relative to the null distribution. For each cancer type and regulatory element, the null distribution of overlapping counts was generated by using all 2761 CpGs as the background and randomly sampling the number of differential CpGs. This procedure was repeated 10,000 times, and the number of overlaps was used to determine the null distribution. The fold-change was calculated as the number of true overlapping counts divided by the mean of the null distribution. Darker green represents a higher enrichment fold (*p*-value < 0.05).

The distribution of the relative positions of differential CpG sites and their corresponding genes are shown in Figure S3 for each cancer type. The distribution of differential CpGs generally follows the distribution of all 2761 tested CpGs. The black line represented the distribution of all CpGs in the test, whereas grey bars showed the differential CpGs with FDR < 0.05. Most of the differential CpGs were located in promoter regions surrounding transcription start sites (TSS). To explore the possible mechanisms underlying differential cytosine modifications, we characterized the co-localization of differential CpGs with the ENCODE regulatory elements. The null distribution of overlapping counts was generated by randomly sampling (e.g., the number of differential CpGs from 2761 CpGs for each cancer type) 10,000 times and counting overlaps with regulatory elements. The log_2_ fold-change was calculated as the observed overlapping counts divided by the mean of the null distribution. The most significant enrichment was found in H3K9me1 for BRCA, HNSC, LUAD, and UCEC (hypergeometric test *p*-value < 0.05; Figure 5).

## 3. Discussion

Elucidating epigenetic regulatory factors associated with centrosome genes could enhance an understanding of human cancers. Previous studies were focused on the over- or under-expression of centrosomal genes involved in controlling the centriole structure, such as *CPAP*/*SAS-4*, whose upregulation affected the centriole structure in several model systems [25,26]. However, with its critical role in gene regulation and biological functions, DNA methylation has not been comprehensively investigated for centrosome genes in the context of diverse cancer types. This current study thus leveraged the TCGA 450K array data on a variety of human cancers to investigate the contribution of DNA methylation in centrosome-related genes to cancers, with the goal to develop CpG methylation-based models that can separate different cancer types.

Our findings suggested that tissue-specific CpG methylation underlies cancer type specificity, consistent with previous findings that methylation is important for tissue-specific gene regulation [27]. Although some of the CpGs showed significant associations with gene expressions, we did not observe any significant differences in the mean correlations between tumor and normal samples. The distribution of the differential CpGs were similar to the distribution of all 2761 tested CpGs. Since the cytosine modification at the promoter region is known to suppress gene expression [27], the CpGs located at the gene body may have offset the mean correlation to the positive direction. Moreover, our findings suggested that in tumor samples, the correlation pattern between promoter methylation and gene expression was rewired compared to the normal tissues, indicating that the rewiring of epigenetic regulatory relationships likely contributes to cancer biology.

Notably, cancer-specific models were built for each cancer type using the cancer-specific CpGs related to centrosomes. In general, the centrosome gene-based models showed a significant outperformance for their target cancer type, thus providing further evidence that the centrosome-related CpGs hold the promise of not only being a sensitive cancer biomarker, but also a biomarker that can distinguish different cancer types, which is a great challenge for the existing multi-cancer detection approaches. In addition, a clear enrichment with H3K9me1 in BRCA, HNSC, LUAD, and UCEC was observed. H3K9me1 has been reported to be co-localized with more active promoters surrounding the TSS [28] and is associated with transcriptional activation. These findings suggested that the cytosine modifications in centrosome-related genes may interact with active cis-regulatory elements.

Regarding public health implications, according to the National Cancer Institute: In 2016, an estimated 1.7 million new cases of cancer will be diagnosed in the United States, and 0.6 million people will die from the disease. Unlike genetic variation, which is static through the life course, environmental factors and human behaviors may induce changes in DNA methylation. Therefore, epigenetic changes may serve as mediating factors in the pathway through which environmental factors lead to disease development [29]. More importantly, these changes can also be taken as targets for modification through preventive and therapeutic interventions. Thus, the findings from this study hold the potential to identify a novel class of epigenetic biomarkers for early cancer detection.

We acknowledge that there are several limitations in the current study. First, TCGA only have limited normal samples compared to tumor samples, which leads to an unbalanced sample size and could affect the statistical power and Type I error rate. The FDR procedure was used to address this issue. Second, the 450K array utilizes the bisulfite conversion method to detect the cytosine modification. However, this approach cannot distinguish 5-methylcytosine (5mC) from 5-Hydroxymethylcytosine (5hmC), which could have different biological implications. Future studies using TAB-array or 5hmC-specific approaches could help address this issue [30,31]. Finally, the 450K array has limited coverage in centrosomes due to large arrays of tandemly repeated DNA sequences present in chromosomes. With the recent development of the complete genomic and epigenetic maps of human centromeres [32], future studies utilizing a long-read sequencing approach could further expand our understanding of centrosomes in cancers.

## 4. Materials and Methods

### 4.1. TCGA Cancer Types

In total, ~4000 tumor samples and ~500 normal controls that covered 11 major cancer types from TCGA were analyzed in the current study (Table 1), including 418 bladder urothelial carcinomas (BLCA) with 21 normal tissues, 792 breast invasive carcinomas (BRCA) with 97 normal tissues, 312 colon adenocarcinomas (COAD) with 38 normal tissues, 140 glioblastoma multiforme (GBM) with 2 normal tissues, 528 head and neck squamous cell carcinomas (HNSC) with 50 normal tissues, 324 kidney renal papillary cell carcinomas (KIRC) with 160 normal tissues, 473 lung adenocarcinomas (LUAD) with 32 normal tissues, 370 lung squamous cell carcinomas (LUSC) with 42 normal tissues, 184 primary pancreatic adenocarcinomas (PAAD) with 9 matched normal solid tissues, 98 rectum adenocarcinomas (READ) with 7 normal tissues, and 438 uterine corpus endometrial carcinomas (UCEC) with 46 normal tissues.

### 4.2. TCGA 450K Array Data

Publicly available TCGA DNA methylation was downloaded and aggregated at the GDC Legacy Archive (https://portal.gdc.cancer.gov/; accessed on 11 November 2021). The centrosome-related gene list was based on Jakobsen et al. [5], with a total of 160 centrosome-related genes (Table S1). CpG loci within +/− 10 kb from these 160 genes of interest were included for examination. A total of 3212 CpG sites were included using this approach. We further removed those CpG probes that: (i) ambiguously mapped to the human genome [33]; (ii) contained common SNPs (single nucleotide polymorphisms) if the SNPs located within 20 bps from interrogated CpG sites had MAF (minor allele frequency) >0.01 (based on dbSNP v135) [34]; (iii) had missing data across over 50% of the samples. The final dataset was comprised of 2761 highly reliable, autosomal CpG sites. The M-value, defined as the log_2_ ratio of the intensities of methylated probes versus unmethylated probes [35], was summarized for each CpG site in each individual. Since TCGA only have limited normal samples for each cancer type, in the following analysis, cancer types with less than 30 normal samples were excluded. The final analysis set of the current study comprised 6 cancer types, i.e., BRCA, HNSC, KIRC, LUAD, LUSC, and UCEC.

### 4.3. TCGA RNA-seq Data

The publicly available RNA-seq data was obtained from GDC using the *TCGAbiolinks* R package [36]. Only the primary solid tumor and solid tissue normal data was downloaded. Searching for those samples with both RNA-seq and DNA methylation data resulted in 781 tumors and 84 normal controls for BRCA, 520 tumors and 20 normal controls for HNSC, 318 tumors and 24 normal controls for KIRC, 454 tumors and 21 normal controls for LUAD, 370 tumors and 8 normal controls for LUSC, and 172 tumors and 24 normal controls for UCEC samples (Table 1). Due to missing data, 139 out of 160 centrosome-related genes were retained for further analysis.

### 4.4. Identify Cancer-Specific CpGs

To detect differentially modified CpGs between tumor and normal in each cancer type, the *limma* R package [37] was used to fit a linear model to the DNA methylation data for each CpG probe. False Discovery Rates (FDRs) were estimated using the Benjamini–Hochberg (BH) method [38]. A result with FDR <0.05 was considered significant. To get the cancer-specific differential CpGs, differential CpGs obtained from the previous step were compared across all cancer types, and the ones unique to a specific cancer type were defined as cancer-specific CpGs. In order to find whether cancer-specific cytosine modifications were caused by tissue-specific cytosine modifications, a principal component analysis was carried out for cancer-specific CpGs in normal samples. Figure 2 shows the workflow for identifying cancer-specific CpGs using breast cancer as an example.

### 4.5. Development of a Cancer-Specific Epigenetic Model

The support vector machine (SVM) is a binary classification algorithm [24]. The main idea of SVM is to find a linear decision surface (hyperplane) that can separate patients’ classes and has the largest distance, i.e., largest gap or margin between border-line patients (i.e., support vectors). Specifically, the SVM was used to build a cancer-specific model to distinguish different cancer types. The R package *e1071* was used in the model training [39]. Both linear and Gaussian kernels are used for the model training. The linear SVM classifier can be formulated as follows, by solving an optimization problem over $\;\alpha_{i}$:

$$f\left(x\right)=\overset{N}{\underset{i}{\sum }}\alpha_{i}y_{i}\left(x_{i}^{T}x\right)+b$$

The SVM classifier with the Gaussian kernel is formulated as follows:

$$f\left(x\right)=\overset{N}{\underset{i}{\sum }}\alpha_{i}y_{i}\exp(-|{\left|x-x_{i}|\right|}^{2}/2\sigma^{2})+b$$

In order to avoid an over-fitting problem for model training in this section, 80% of randomly selected samples were used for training purposes. Cancer-specific CpGs with log_2_ fold change >1 and *p*-value < 0.0005 were used as features. If these criteria were not met, the top 5 CpGs with the smallest *p*-values were used. A five-fold cross validation was used during the training process to get the best performance. After the model was trained, the remaining 20% of the samples were used to test the model performance, and the area under the receiver operating characteristic (ROC) curve (AUC) was calculated. The model for each cancer was also tested in other cancer data, in order to prove cancer specificity.

### 4.6. Linking DNA Methylation and Gene Expression

To detect the association between the methylation levels of cancer-specific CpGs and gene expression phenotypes, correlations between the methylation levels and corresponding 139 gene expressions in tumor samples were evaluated. A linear model was fitted for each CpG with its corresponding gene for each cancer type separately: i.e., $G\;\sim \;\beta_{1}M+\beta_{2}gender+e$; where M is the methylation level of CpG and G is the expression level of the corresponding gene. An FDR of less than 0.05 estimated using the BH procedure was considered significant.

### 4.7. Co-localization of Differential CpGs with Cis-Regulatory Elements

We obtained uniformly processed narrow peaks for transcription factor binding sites and broad peaks for histone markers from the ENCODE (Encyclopedia of DNA Elements) Project [40]. Peaks for each of the canonical transcription factors and histone modification markers were examined individually. We mapped all analyzed CpG sites to positional bins including 2 kb bins along the upstream 10 kb from the transcriptional start site (TSS), 10 percentile-bin along the coding region, 2 kb bins along the downstream 10 kb from the transcriptional end site (TES). To estimate the null distributions for ENCODE co-localization, we used all 2761 CpGs as background, randomly sampled the number of differential CpGs 10,000 times, mapped to the peaks of regulatory elements in the same manner as differential CpGs, and counted the number of CpG sites co-localized with the peaks for the given marker. The number of true co-localizations were then compared with the null distribution, and the log_2_ fold-change was calculated as:

$$log_{2}\left(fold-change\right)=\frac{number\;of\;true\;overlaps}{mean\;\left(null\;distribution\right)}$$

## 5. Conclusions

In conclusion, by utilizing the TCGA data, this work explored the distinct role of cytosine modifications for centrosome-related genes and revealed the cancer-specific cytosine modification patterns. Furthermore, this cancer-specific pattern of epigenetic modification demonstrated its potential as a novel cancer biomarker that may aid in diagnosis and targeted screenings for at-risk individuals.

## Figures and Tables

**Table 1:** The analyzed set of TCGA samples.

Cancer	# Tumor Samples	# Normal Samples	# Tumor Samples	# Normal Samples	Cancer Type
	450K Array		RNA-seq		
BLCA	418	21			Bladder urothelial carcinomas
BRCA	792	97	781	84	Breast invasive carcinomas
COAD	312	28			Colon adenocarcinomas
GBM	140	2			Glioblastoma multiformes
HNSC	528	50	520	20	Head and neck squamous cell carcinomas
KIRC	324	160	318	24	Kidney renal papillary cell carcinomas
LUAD	473	32	454	21	Lung adenocarcinomas
LUSC	370	42	370	8	Lung squamous cell carcinomas
PAAD	184	10			Pancreatic adenocarcinomas
READ	98	7			Rectum adenocarcinomas
UCEC	438	46	172	24	Uterine corpus endometrial carcinomas
**Total**	**4077**	**505**	2615	181	

**Table 2:** Summary of the detected and cancer-specific differential CpGs.

	# Differential CpGs (FDR < 0.05)	# Hosting Genes ^a^	# Cancer-Specific CpGs	# Hosting Genes ^b^	Cancer-Specific Genes
BRCA	701	123	89	54	*CETN2*, *MAP7D3*
HNSC	531	121	86	62	-
KIRC	1283	133	186	88	*PRKAR2B*, *CEP290*, *NOG*, *CDK5RAP2*, *HAUS6*, *PRKACB*, *PRKAR2A*, *NPHP4*
LUAD	228	87	9	5	-
LUSC	1366	136	320	110	*RTTN*, *DCTN5*, *CEP120*, *IRAK1BP1*, *SSNA1*, *CEP135*, *ACTR1A*, *PCM1*
UCEC	489	112	45	37	*ODF2*, *DCTN3*, *PIBF1*

^a^ Corresponding gene for the differential CpGs. ^b^ Corresponding gene for the cancer-specific CpGs. FDR: false discovery rate.

## Data Availability

The data that support the findings of this study are available from the corresponding author upon reasonable request.

## Supplementary Materials

The following supporting information can be downloaded at: https://www.mdpi.com/article/10.3390/epigenomes6020014/s1, Figure S1. Correlation of CpG methylation and gene expression; Figure S2. Correlation of CpG methylation and gene expressions by genomic feature; Figure S3. Relative distribution of differential CpGs; Table S1. The analyzed centrosome-related genes.
