# Supplementary material for: Epigenetic Signatures of Centrosomes Are Novel Targets in Cancer Diagnosis: Insights from an Analysis of the Cancer Genome Atlas
Source: Epigenomes. 2022 Jun 2;6(2):14. doi: 10.3390/epigenomes6020014 (PMC9222712; doi:10.3390/epigenomes6020014)
Supplement: Supplementary file 1 [file epigenomes-06-00014-s001.zip › epigenomes-1733575-supplementary.pdf]

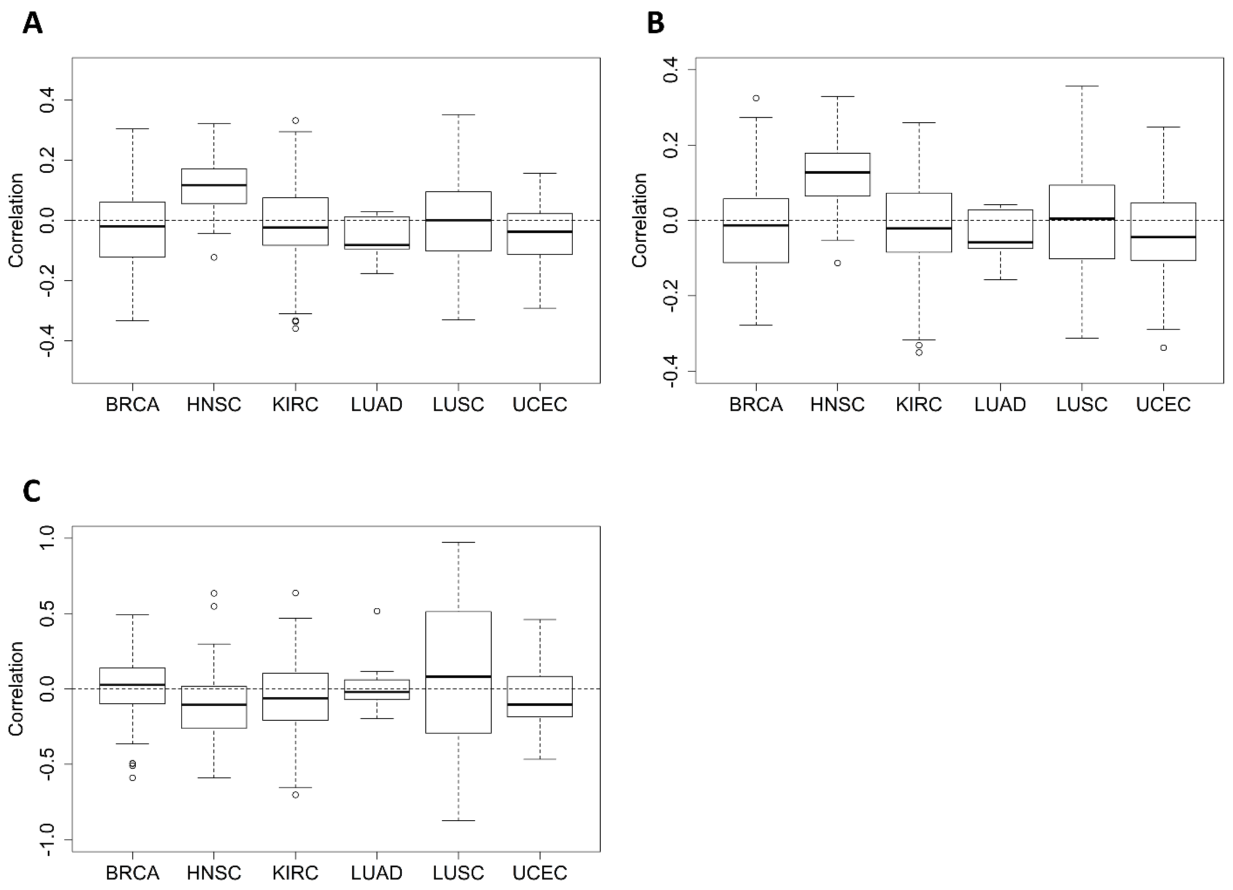

Figure S1. Correlation of CpG methylation and gene expression.

A) Tumor and normal samples combined. The mean correlations are -0.02 (BRCA), 0.11 (HNSC), -0.01 (KIRC), -0.06 (LUAD), 0 (LUSC), and -0.04 (UCEC); (B) Tumor samples only. The mean correlations are 0.02 (BCRA), 0.12 (HNSC), -0.01 (KIRC), -0.37 (LUAD), 0 (LUSC), and -0.03 (UCEC); (C) Normal samples only. The mean correlations 0.01 (BCRA), -0.12 (HNSC), -0.05 (KIRC), 0.02 (LUAD), 0.09 (LUSC), and -0.04 (UCEC).

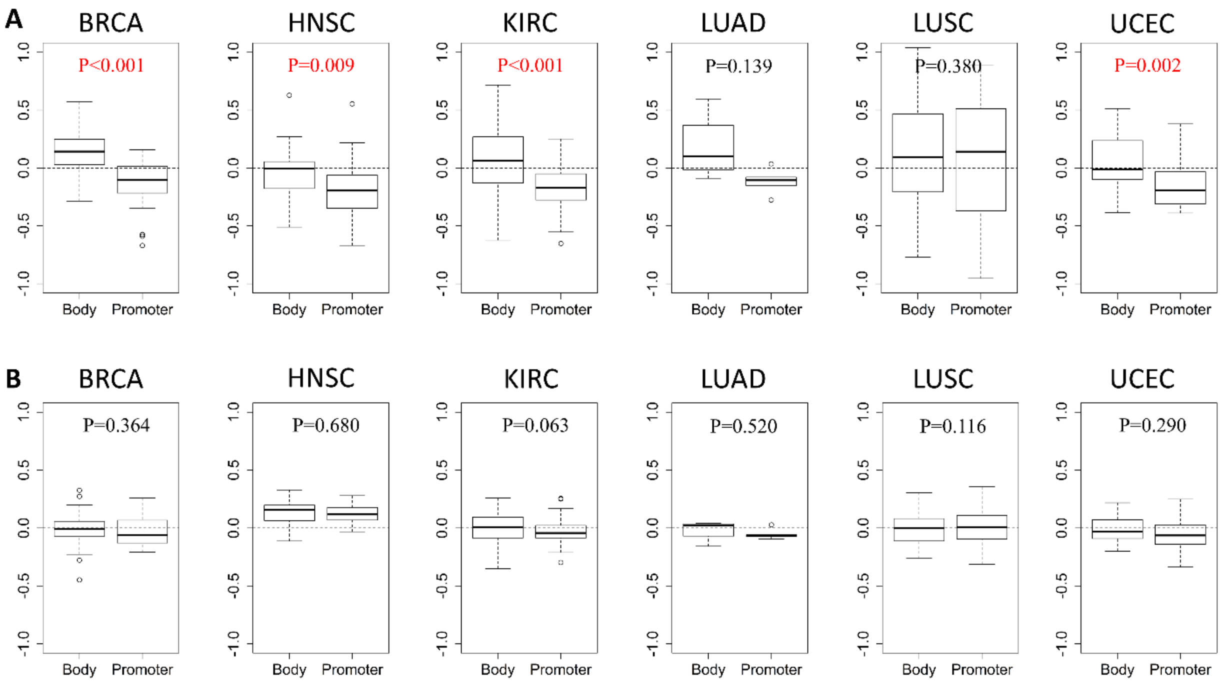

Figure S2. Correlation of CpG methylation and gene expressions by genomic feature.
Each boxplot shows the correlations between the cytosine modifications of differential CpGs and corresponding gene expressions at gene body or promoter region. (A) The correlations in normal samples for BCRA, HNSC, KIRC, LUAD, LUSC, and UCEC, respectively; (B) The correlations in tumor samples for BCRA, HNSC, KIRC, LUAD, LUSC, and UCEC, respectively. Significant p-values are highlighted in red.

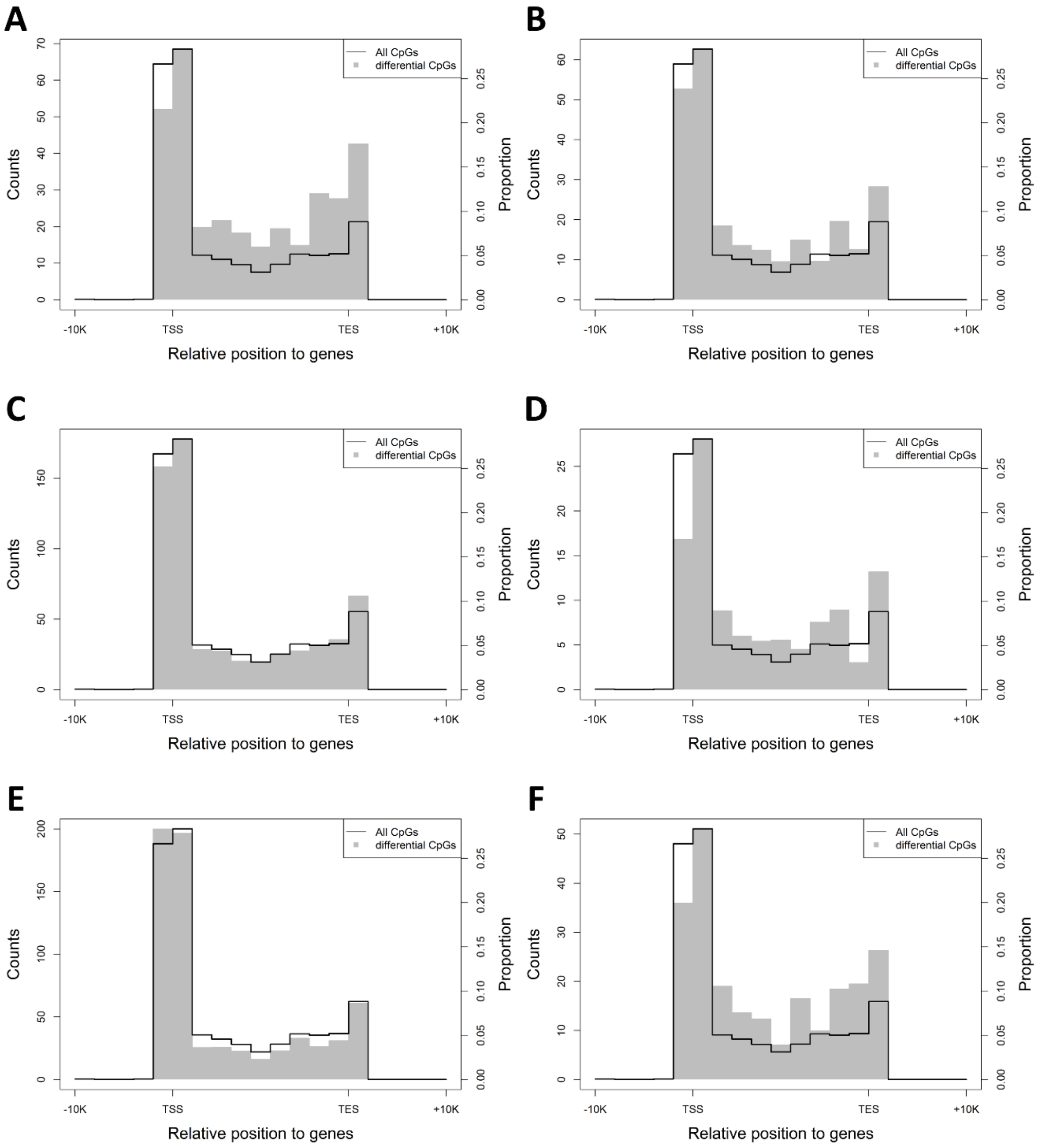

Figure S3. Relative distribution of differential CpGs.

The CpG sites were mapped to the 5kb bins up-/down-stream of TSS and TES, respectively, as well as 10 percentile bins along the gene body. The black line shows the distribution of all 2761 CpGs in the test, while grey bars represent the differential CpGs with FDR<0.05.

Table S1. The analyzed centrosome-related genes.

ENSEMBL ID	Gene symbol	ENSEMBL ID	Gene symbol
ENSG00000005249	PRKAR2B	ENSG00000136811	ODF2
ENSG00000007168	PAFAH1B1	ENSG00000136861	CDK5RAP2
ENSG00000011523	CEP68	ENSG00000137100	DCTN3
ENSG00000046651	OFD1	ENSG00000137601	NEK1
ENSG00000051825	MPHOSPH9	ENSG00000137814	HAUS2
ENSG00000054282	SDCCAG8	ENSG00000137822	TUBGCP4
ENSG00000058453	CROCC	ENSG00000138107	ACTR1A
ENSG00000068796	KIF2A	ENSG00000138180	CEP55
ENSG00000072062	PRKACA	ENSG00000138686	BBS7
ENSG00000072803	FBXW11	ENSG00000139323	POC1B
ENSG00000072864	NDE1	ENSG00000139350	NEDD1
ENSG00000074054	CLASP1	ENSG00000139437	TCHP
ENSG00000077380	DYNC1I2	ENSG00000139737	SLAIN1
ENSG00000078674	PCM1	ENSG00000141551	CSNK1D
ENSG00000079335	CDC14A	ENSG00000141577	AZI1
ENSG00000082512	TRAF5	ENSG00000141933	TPGS1
ENSG00000082701	GSK3B	ENSG00000142731	PLK4
ENSG00000083535	PIBF1	ENSG00000142875	PRKACB
ENSG00000087586	AURKA	ENSG00000143156	NME7
ENSG00000088986	DYNLL1	ENSG00000143702	CEP170
ENSG00000090615	GOLGA3	ENSG00000144635	DYNC1LI1
ENSG00000092020	PPP2R3C	ENSG00000146243	IRAK1BP1
ENSG00000092036	HAUS4	ENSG00000147400	CETN2
ENSG00000100503	NIN	ENSG00000147874	HAUS6
ENSG00000100578	KIAA0586	ENSG00000148019	CEP78
ENSG00000100629	CEP128	ENSG00000149548	CCDC15
ENSG00000101367	MAPRE1	ENSG00000151466	SCLT1
ENSG00000101624	CEP76	ENSG00000151849	CENPJ
ENSG00000101639	CEP192	ENSG00000152082	MZT2B
ENSG00000101752	MIB1	ENSG00000152240	HAUS1
ENSG00000103494	RPGRIP1L	ENSG00000152359	POC5
ENSG00000103540	CCP110	ENSG00000153140	CETN3
ENSG00000103995	CEP152	ENSG00000153575	TUBGCP5
ENSG00000104218	CSPP1	ENSG00000154240	CEP112
ENSG00000104671	DCTN6	ENSG00000154429	CCSAP
ENSG00000104983	CCDC61	ENSG00000156787	TBC1D31
ENSG00000106477	CEP41	ENSG00000156876	SASS6
ENSG00000106948	AKNA	ENSG00000157456	CCNB2
ENSG00000107890	ANKRD26	ENSG00000160299	PCNT
ENSG00000109171	SLAIN2	ENSG00000160410	SHKBP1
ENSG00000109881	CCDC34	ENSG00000160813	PPP1R35
ENSG00000110274	CEP164	ENSG00000161996	WDR90
ENSG00000111554	MDM1	ENSG00000163611	SPICE1

ENSG00000111860	CEP85L
ENSG00000112877	CEP72
ENSG00000114107	CEP70
ENSG00000114302	PRKAR2A
ENSG00000115073	ACTR1B
ENSG00000116127	ALMS1
ENSG00000116198	CEP104
ENSG00000116213	WRAP73
ENSG00000116691	MIIP
ENSG00000117650	NEK2
ENSG00000119397	CNTRL
ENSG00000119685	TTLL5
ENSG00000120647	CCDC77
ENSG00000121289	CEP89
ENSG00000122417	ODF2L
ENSG00000123473	STIL
ENSG00000126001	CEP250
ENSG00000126216	TUBGCP3
ENSG00000127914	AKAP9
ENSG00000128159	TUBGCP6
ENSG00000129680	MAP7D3
ENSG00000130640	TUBGCP2
ENSG00000130695	CEP85
ENSG00000130779	CLIP1
ENSG00000131351	HAUS8
ENSG00000131462	TUBG1
ENSG00000131697	NPHP4
ENSG00000131966	ACTR10
ENSG00000132359	RAP1GAP2
ENSG00000132912	DCTN4
ENSG00000133393	FOPNL
ENSG00000133739	LRRCC1
ENSG00000135315	KIAA1009
ENSG00000135720	DYNC1LI2
ENSG00000135736	CCDC102A
ENSG00000135837	CEP350
ENSG00000136108	CKAP2
ENSG00000164087	POC1A
ENSG00000164118	CEP44
ENSG00000166004	KIAA1731
ENSG00000166037	CEP57
ENSG00000166847	DCTN5
ENSG00000166851	PLK1
ENSG00000168014	C2CD3
ENSG00000168944	CEP120
ENSG00000169607	CKAP2L
ENSG00000169683	LRRC45
ENSG00000169991	IFFO2
ENSG00000170037	CNTROB
ENSG00000170264	FAM161A
ENSG00000173226	IQCB1
ENSG00000173272	MZT2A
ENSG00000173588	CCDC41
ENSG00000174007	CEP19
ENSG00000174799	CEP135
ENSG00000175203	DCTN2
ENSG00000175216	CKAP5
ENSG00000175455	CCDC14
ENSG00000176101	SSNA1
ENSG00000176225	RTTN
ENSG00000178104	PDE4DIP
ENSG00000182504	CEP97
ENSG00000182923	CEP63
ENSG00000183137	CEP57L1
ENSG00000183691	NOG
ENSG00000188878	FBF1
ENSG00000197102	DYNC1H1
ENSG00000198331	HYLS1
ENSG00000198707	CEP290
ENSG00000198920	KIAA0753
ENSG00000204843	DCTN1
ENSG00000213066	FGFR1OP
ENSG00000213397	HAUS7
ENSG00000214367	HAUS3
